# An Attenuated Herpes Simplex Virus Type 1 (HSV1) Encoding the HIV-1 Tat Protein Protects Mice from a Deadly Mucosal HSV1 Challenge

**DOI:** 10.1371/journal.pone.0100844

**Published:** 2014-07-17

**Authors:** Mariaconcetta Sicurella, Francesco Nicoli, Eleonora Gallerani, Ilaria Volpi, Elena Berto, Valentina Finessi, Federica Destro, Roberto Manservigi, Aurelio Cafaro, Barbara Ensoli, Antonella Caputo, Riccardo Gavioli, Peggy C. Marconi

**Affiliations:** 1 Department of Life Sciences and Biotechnology, Section of Applied Microbiology and Pathology, University of Ferrara, Ferrara, Italy; 2 Department of Life Sciences and Biotechnology, Section of Biochemistry and Molecular Biology, University of Ferrara, Ferrara, Italy; 3 Department of Molecular Medicine, University of Padova, Padova, Italy; 4 National AIDS Center, Istituto Superiore di Sanità, Rome, Italy; UC Irvine Medical Center, United States of America

## Abstract

Herpes simplex virus types 1 and 2 (HSV1 and HSV2) are common infectious agents in both industrialized and developing countries. They cause recurrent asymptomatic and/or symptomatic infections, and life-threatening diseases and death in newborns and immunocompromised patients. Current treatment for HSV relies on antiviral medications, which can halt the symptomatic diseases but cannot prevent the shedding that occurs in asymptomatic patients or, consequently, the spread of the viruses. Therefore, prevention rather than treatment of HSV infections has long been an area of intense research, but thus far effective anti-HSV vaccines still remain elusive. One of the key hurdles to overcome in anti-HSV vaccine development is the identification and effective use of strategies that promote the emergence of Th1-type immune responses against a wide range of epitopes involved in the control of viral replication. Since the HIV1 Tat protein has several immunomodulatory activities and increases CTL recognition of dominant and subdominant epitopes of heterologous antigens, we generated and assayed a recombinant attenuated replication-competent HSV1 vector containing the *tat* gene (HSV1-Tat). In this proof-of-concept study we show that immunization with this vector conferred protection in 100% of mice challenged intravaginally with a lethal dose of wild-type HSV1. We demonstrate that the presence of Tat within the recombinant virus increased and broadened Th1-like and CTL responses against HSV-derived T-cell epitopes and elicited in most immunized mice detectable IgG responses. In sharp contrast, a similarly attenuated HSV1 recombinant vector without Tat (HSV1-LacZ), induced low and different T cell responses, no measurable antibody responses and did not protect mice against the wild-type HSV1 challenge. These findings strongly suggest that recombinant HSV1 vectors expressing Tat merit further investigation for their potential to prevent and/or contain HSV1 infection and dissemination.

## Introduction

Worldwide prevalence of the herpes simplex virus (HSV) infection remains high, making it a major public health concern. Indeed, HSV type 1 (HSV1) and type 2 (HSV2) are pathogens well-adapted to their human hosts, infecting them through lytic infection of cutaneous and mucosal epithelial cells, and can lie dormant in the sensory ganglia, reactivating periodically [1]. Recurrent productive infections, which can be either symptomatic or asymptomatic (and therefore unwittingly spread), give rise to several clinical illnesses, including cold sores, keratitis, blepharitis, meningitis, encephalitis and genital infections, which may have severe sequelae in neonatal and immune-compromised patients [2]–[7].

Due to unwitting transmission, latent infection, periodic reactivation and asymptomatic virus shedding, HSV is easily spread and is unlikely to be eradicated by preventative strategies. Indeed, currently available drugs are only efficacious against replicating HSV, but have no effect on the latent virus or its reactivation [8]. Hence the identification of new vaccination approaches capable of preventing the spread of the virus and/or blocking its reactivation is likely to have great global impact on public health. Unfortunately, however, the numerous attempts to develop anti-HSV vaccines have thus far proved unsuccessful [9]–[20]. Chiron and GlaxoSmithKline vaccine candidates based on recombinant HSV envelope glycoproteins have failed to show efficacy [21], [22]. This has prompted researchers to increase their efforts to define immune correlates of protection and new vaccination strategies able to induce protective immunity [8], [19], [20], [23]. Recent evidence strongly suggests that specific cellular immune responses are key for HSV control in humans, in particular those directed against “asymptomatic” CD8^+^ epitopes [24], which appear to mediate protection in asymptomatic HSV-infected individuals [24]–[27]. It seems likely therefore that the effectiveness of HSV vaccines may depend on their capacity to induce cellular immune responses against specific subsets of viral epitopes for which correct antigen presentation is an essential prerequisite [28], [29]. Thus, the use of molecules favoring the emergence of Th1 immune responses against such epitopes could feasibly represent a relevant avenue for anti-HSV vaccine research [25], [30]–[34]. However, although several molecules have been reported to enhance Th1-type responses, agents able to induce class I-restricted CTL responses directed against subdominant epitopes have not yet been identified, with the exception of a recently described cytomegalovirus vector approach [35]. In search for new vaccination strategies capable of fighting HSV infection and disease, we investigate whether a live attenuated HSV1-derived vector expressing the HIV-1 Tat protein (HSV1-Tat) could elicit broad protective immunity against HSV. Indeed, previous *in vitro* (B, T and dendritic cells) and *in vivo* (mice, non-human primates and humans) evidence indicates that the Tat protein, in addition to being a safe and relevant HIV vaccine antigen, possesses several immunomodulatory features that could make it suitable for new vaccination strategies and therapeutic interventions aimed at modulating antigen-specific immune responses in various human diseases [36]. In particular, biologically active clade-B Tat protein (aa 1–86) very actively targets immature dendritic cells, inducing their maturation and polarizing the immune response to the Th1 pattern through transcriptional activation of TNF-alpha gene expression, leading to a more efficient presentation of both allogeneic and heterologous antigens [37], [38]. Tat also induces changes in the subunit composition of the immune proteasome that result in altered enzyme activities and modulation of CTL epitope generation in virally-infected cells, broadening *in vivo* T-cell responses against cryptic epitopes of a co-antigen (otherwise not expressed, or only poorly expressed) [39]–[41]. In addition, Tat possesses an intrinsic adjuvanticity, attributed to its dimerization capacity [42], increases the number of regulatory T-cells (Treg) [43], promotes the activation of virus-specific CTLs [44], and induces protective immunity against *Leishmania major* [45].

Recombinant HSV-based vectors, either live attenuated or non-replicating, are considered promising vaccine candidates against HSV infections [46], [47]. Of particular relevance to the use of live attenuated HSV-based vectors are the recent observations regarding the ability of HSV to induce antibody and proliferative responses, regardless of pre-existing immunity to HSV [48], [49]. They have also yielded satisfactory safety results in independent preclinical and clinical studies for cancer gene therapy [47] (http://clinicaltrials.gov/). At this stage, this makes live attenuated viruses valid research targets, despite their inherent risks. Hence, we decided to investigate the local and systemic effects of a recombinant live-attenuated HSV1-Tat vector after intravaginal challenge with wild-type HSV1. Interestingly, we found that although not all mice immunized with the recombinant live-attenuated HSV1-Tat vector displayed antibody responses against HSV1, 100% of them developed cellular immune responses and were protected against a challenge with a lethal dose of wild-type HSV1. As neither of these effects were induced by the control viral vector without Tat (HSV1-LacZ), it appears that recombinant HSV1 vectors expressing Tat merit further investigation in the field of anti-HSV vaccination strategies.

## Materials and Methods

### Ethics statement

All animal experiments were conducted in conformity to European and Institutional guidelines for the housing and care of laboratory animals and performed under protocols approved by the Italian Ministry of Health.

### Cell lines

Vero cells (African green monkey kidney cell line, ATCC), 3T3 BALB/c cells, a fibroblast cell line derived from BALB/c mice, and human HeLa3T1 cells [50], containing an integrated copy of plasmid HIV-LTR-CAT in which expression of the chloramphenicol acetyl transferase (CAT) reporter gene is driven by the HIV-1 LTR promoter, were grown in DMEM (Euroclone, Grand Island, NY). The mouse dendritic cell line CB1 [51] was grown in ISCOV (Euroclone). Media were supplemented with 10% FBS (Euroclone), 1% L-glutamine (100X solution, 0.2 M, BioWhittaker, Walkersville, MD), 1% penicillin/streptomycin (100X solution, Euroclone). The cells were detached with trypsin solution containing 0.25% trypsin and 0.02% EDTA (Euroclone). Splenocytes from immunized and control mice were cultured in RPMI 1640 (Euroclone) supplemented with 10% Hyclone (Euroclone), 50 µM β-mercaptoethanol (Gibco, Grand Island, NY), 1% L-glutamine, 1% penicillin/streptomycin, 1% non-essential aminoacids (Sigma-Aldrich, St. Louis, MO) and 1 mM sodium pyruvate (Sigma-Aldrich) (referred to as RPMI1640 complete medium). All cells were grown at 37°C in 5% CO_2_.

### Plasmids and genes

Plasmids and genes used were: pTZ18U (Bio-rad, Glen Allen, VA, USA), pBlueScript SK (Stratagene, California, USA), pcDNA 3.1/Hygro^(+)^ (Invitrogene, Life Technologies, Paisley, UK), EGFP gene from pIRES-GFP (Clontech, Laboratories Inc., Mountain View, USA), and *lacZ* gene from pβgal-Basic vector (BD Biosciences, Becton Dickinson, USA) whose sequence is available in GenBank (accession number U13184). The pCV-tat expressing the HIV-1 tat cDNA (HTLV-IIIB isolate, subtype B) has previously been described by Arya and co-workers [52], and the plasmid pB41-LacZ, in which the *lacZ* gene is surrounded by HSV UL41 flank sequences, by Krisky and co-workers [53], [54]. All HSV1 gene coordinates used throughout this study were determined on the basis of a sequence available in GenBank (accession number NC_001806).

### Generation of plasmids and attenuated replication-competent HSV1-based vectors

The plasmid pB41-lacZ contains the *lacZ* coding sequence (flanked at 5′ and 3′ ends by Pac I sites) inserted into the UL41 locus of HSV1 between UL41 flank fragments (HSV genomic positions 90145–91631 and 92230–93854 and Sma 599 bp deletion 91631–92230 UL41) under the control of HSV1 ICP0 promoter (ICP0pr, StuI-DrdI fragment, sequence available at GenBank NC_001806), as previously described [53], [54]. The recombinant attenuated replication-competent HSV1-LacZ, in which the UL41 gene is deleted by insertion of the *lacZ* gene under the control of the HSV ICP0pr, was generated by homologous recombination between wild-type HSV1 strain LV [55] and plasmid pB41-lacZ. Briefly, Vero cells were co-transfected with the DNA from HSV1 (strain LV [55]) and from plasmid pB41-lacZ at different concentration ratios. The recombinant HSV1-LacZ virus was identified by isolation of cells with a blue plaque phenotype after X-gal staining. To this end, cells were fixed with 1.5% glutaraldehyde in PBS, washed three times with PBS, and then incubated in the dark at 37°C with X-gal stain (Sigma-Aldrich), according to the manufacturer’s instructions.

The *tat* cDNA (350 bp) was obtained from pCV-tat [52] following digestion with PstI, and then ligated into the PstI site of plasmid pTZ18U to generate plasmid pTZ18U-Tat. The plasmid pB41-tat, containing the *tat* cDNA inserted into the UL41 locus of HSV1 under the control of HSV-1 ICP0pr, was obtained from pB41-lacZ by replacing *lacZ* coding sequences with *tat* cDNA. Briefly, *tat* cDNA was obtained from pTZ18U-Tat following digestion with HindIII-blunted/XbaI, and inserted into EcoRI-blunted/XbaI sites of the pB41-lacZ plasmid. Finally, the HSV1-ICP0pr was substituted with the HCMV promoter derived from the commercial vector pcDNA3.1, following digestion with NruI/PmeI (both are blunt-end sites) and insertion into the SmaI site of plasmid pB41-tat to generate vector pB41-HCMVtat. The recombinant live attenuated HSV1-Tat vector was constructed by means of homologous recombination between UL41 sequences of the pB41-HCMVtat plasmid and the HSV1-LacZ vector, using the previously described Pac-facilitated *lacZ*-substitution method [53], [54]. Briefly, Vero cells were co-transfected with the HSV1-LacZ viral DNA cleaved with PacI (in order to excide lacZ) and the pB41-HCMVtat plasmid DNA, linearized with NotI, at different concentration ratios. The recombinant HSV1-Tat was then identified by isolation of cells with a clear plaque phenotype after X-gal staining, performed as described above.

The HSV1-LacZ and HSV1-Tat viruses were purified by three rounds of limiting dilution, each followed by Southern blot analysis to confirm the presence of the transgenes *lacZ or tat*. The viral DNAs were isolated from infected cell lysates using 10 mM Tris-HCl (pH 8.0), 10 mM EDTA, 0.6% SDS and proteinase K (0.25 mg/ml) followed by phenol:chloroform:isoamyl alcohol (25∶25∶1) and chloroform:isoamyl alcohol (25∶1) extraction procedures [56]. Aliquots of viral DNA were digested with XbaI overnight at 37°C, fractionated by 0.8% agarose gel electrophoresis, transferred to a Hybond-N+ nylon membrane (Amersham Pharmacia, Little Chalfont, UK), and hybridized with an HSV1 HindIII-SmaI fragment spanning the 3′ end of the HSV1-UL41 (vhs) coding sequence, *lacZ* or *tat* DNA sequences (*lacZ* fragment EcoV-NdeI from pβgal-Basic vector or *tat* PstI fragment from pCV-tat) using the ECL Direct Nucleic Acid Labeling and Detection Systems Kit (Amersham Pharmacia) according to the manufacturer’s instructions.

### Large-scale virus stock purification

HSV1-Tat, HSV1-LacZ and wild-type HSV (HSV1 LV) stocks were prepared by infecting Vero cells (4×10^8^) in suspension with each recombinant virus at a MOI (multiplicity of infection) of 0.05 plaque-forming units (pfu)/cell for 1 h at 37°C under mild agitation. The viral inoculum was then removed, and infected cells were seeded into the 150–175 cm^2^ flasks, cultured at 37°C until a 100% cytopathic effect was evident [56], and collected by centrifugation at 2,500 rpm (*1,204*×*g*) for 15 min at 4°C. Supernatants were spun at 20,000 rpm (*48,384*×*g)* at 4°C in a JA20 rotor (Beckman, Milan, Italy) for 30 min to collect the virus, whereas the cell pellets were resuspended in 2 ml of medium, then subjected to three cycles of freeze–thawing (−80°C/37°C) and a single burst of sonication to release the viral particles. The virus was further purified by density gradient centrifugation (Opti Prep; Invitrogen Life Technologies) and resuspended in PBS without calcium and magnesium (Euroclone). Viral stocks were titered *in vitro* by the plaque assay method [56], as described in the following section, and stored at −80°C.

### Viral yield evaluation and plaque assay

Vero cells (1×10^6^) were infected either in adhesion or in suspension with HSV1-Tat or HSV1-LacZ recombinant vectors at either 0.1 or 1 MOI. Following 1 h adsorption, the infected cells were incubated in 5% CO_2_ for 18 h at 37°C. Samples were then collected, subjected to three cycles of freeze–thawing, sonicated and titrated onto Vero cells to determine the viral yield, as previously described [56]. Briefly, the HSV1-Tat or HSV1-LacZ virus suspensions were added to six-well plates containing 1×10^6^ Vero cells/well at 37°C in 5% CO_2_ for 1 h. Next the viral inoculum was removed and 1.5% methylcellulose overlay medium was added to each well. When plaques appeared in the cell monolayers, the plates were fixed with 2 ml/well of 1% crystal violet solution (50∶50 methanol: dH_2_O v/v) for 20–30 minutes, rinsed with water, and air-dried. Plaques were then counted, and the virus titers were calculated as numbers of pfu/ml.

### Western blot analysis

Tat protein expression from the recombinant vector was analysed in both BALB/c and Vero cells (1×10^6^ cells) infected with HSV1-Tat at 1 MOI. Negative controls were uninfected cells, and cells infected with 1 MOI of the HSV1-LacZ control vector. Cell extracts, corresponding to 10 µg of total proteins, were loaded into 12% SDS-polyacrylamide gels and analysed by Western blot using a rabbit anti-Tat polyclonal serum (Intracel, Cupertino, CA) at 1∶1,000 dilution and mouse anti-rabbit horseradish peroxidase (HRP)-conjugated secondary antibody (Sigma-Aldrich) at 1∶4,000 dilution. Immunocomplexes were detected by means of the ECL Western Blot detection kit (Amersham Pharmacia). A recombinant Tat protein (1 µg), obtained from Diatheva (Urbino, Italy), was included in each gel as positive control.

### CAT assay

An Enzyme-Linked Immune Assay (CAT ELISA) was used to quantify the CAT expression induced by the functional Tat protein produced by the viral vector, using a commercially available kit provided by Roche (Mannheim, Germany). HeLa 3T1 cells (1×10^6^) were infected in suspension with HSV1-Tat and the control vector HSV1-LacZ, at two different MOIs (0.1 or 1), for 1 h at 37°C under mild shaking. After infection, cells were washed twice with complete medium to eliminate any free virus particles, plated onto six-well plates, and cultured at 37°C for 6, 12 or 24 h. After incubation, cells were disrupted with lysis buffer 1X. The cell extracts (containing the CAT enzyme) were then added to microplate wells, pre-coated with an anti-CAT polyclonal antibody according to the manufacturer’s instructions. The absorbance of the sample, a direct correlate of the level of CAT present in the cell extracts, was determined at 405 nm using an ELISA reader.

### Peptides

Peptides were synthesized by the solid-phase method, and purified by HPLC to >98% purity (UF Peptides, Ferrara, Italy). HSV1 K^b^-restricted peptides SSIEFARL (SSI), derived from glycoprotein B (gB) [57], [58]; ITAYGLVL (ITA), derived from glycoprotein K (gK) [59], [60]; and QTFDFGRL (QTF), derived from ribonucleotide reductase 1 (RR1) [61], were used to evaluate anti-HSV1 T-cell responses in C57BL/6 mice. HSV1 K^d^-restricted peptide DYATLGVGV (DYA), derived from ICP27 [62], [63], and SLKMADPNRFRGKDLP (SLK), which includes both the H^b^-restricted CD4 and K^d^-restricted CD8 immunodominant epitopes derived from glycoprotein D (gD) [64]–[66], were used to evaluate anti-HSV1 T-cell responses in BALB/c mice. Peptide stocks were prepared in DMSO at 10^−2^ M concentration, stored at −20°C, and diluted in RPMI 1640 before use.

### *In Vivo* titration of wild-type HSV1 and live attenuated HSV1-Tat or HSV1-LacZ

Animals were handled according to European and Institutional guidelines. The lethal dose (LD) of wild-type HSV1 was determined in both BALB/c and C57BL/6 female mice, since susceptibility to HSV1 infection varies according to mouse gender and strain [67], [68]. To bring the mice to the same oestrous stage and render them more susceptible to HSV infection, 2 mg/100 µl of Depo-Provera (Depo-medroxy-progesterone acetate; Pharmacia & Upjohn, Somerset County, NJ) was injected subcutaneously into the neck of 8-week old female BALB/c and C57BL/6 mice (Charles-River, Lecco, Italy), one week before challenge [69]. Mice (n = 7) were then inoculated intravaginally with a range of 2×10^4^ to 2×10^8^ pfu/mouse (10 µl) of wild-type HSV1, LV strain [55] to determine the lethal doses for challenge experiments. Before injection, the virus was thawed on ice, sonicated for 5 s, and stored on ice. Mice were anaesthetized with 5% isofluorane to allow scraping of the vagina with a pipe cleaner (to remove any mucus, which could trap the virus) and then inoculated with the purified virus using a pipette tip. After infection, mice were observed daily to monitor the appearance of any local and/or systemic clinical signs of infection, including death. Disease severity was measured using the following arbitrary scores: 0 (no signs), 1 (ruffled fur and genital inflammation), 2 (appearance of cold sores on and around the vagina), 3 (paralysis of the hind limbs) and 4 (pre-moribund state and death). Each titration experiment was repeated 3 times. BALB/c mice died at the dose of 2×10^6^ pfu/mouse, whereas C57BL/6 mice succumbed at the dose of 2×10^8^ pfu/mouse. Accordingly, these dosages were used as lethal doses for challenging mice immunized with HSV1-LacZ or HSV1-Tat. Similar titration experiments were performed in BALB/c mice (n = 10) in order to determine infectivity and attenuation of the HSV1-Tat and HSV1-LacZ vectors, at doses ranging from 10^4^ to 10^8^ pfu/mouse administered by the intravaginal route.

### Immunization with HSV1-Tat or HSV1-LacZ recombinants and challenge with wild-type HSV1

To determine the dose of virus capable of inducing optimal anti-HSV1 immune responses, C57BL/6 and BALB/c female mice (n = 7) were treated intravaginally with 10^2^ to 10^4^ pfu/mouse (10 µl) of the recombinant replication-competent HSV1-Tat virus, or with the control virus HSV1-LacZ, as described in the previous paragraph. Mice were sacrificed 15 days later to evaluate anti-HSV1 immune responses, as described below. None of the doses of either vector tested for the immunization studies induced the appearance of local signs of infection, but the lowest dose of HSV1-Tat and HSV1-LacZ capable of inducing detectable cellular immune responses in 100% of treated mice was 10^3^ pfu/mouse, which was therefore chosen as the optimal immunization dose.

In immunization experiments, six-week-old C57BL/6 and BALB/c female were then treated intravaginally with 10^3^ pfu/mouse of HSV1-Tat or with HSV1-LacZ. Each experimental group was composed of 15 animals. Seven days post-infection, mice (n = 5 per experimental group) were sacrificed to evaluate any HSV1-specific T-cell responses on splenocyte cultures (individual mice) by means of IFN-γ and IL-4 ELISpot assays. In 5 mice per experimental group, the presence of HSV1-specific antibodies (IgM, IgG and IgA in sera and IgG and IgA in vaginal washes) was assessed at day 20 post-treatment by enzyme-linked immunoassays (ELISA). At day 28, mice (n = 10 per experimental group) were challenged intravaginally with a lethal dose of wild-type HSV1 strain LV [55] (2×10^6^ pfu/mouse for BALB/c and 2×10^8^ pfu/mouse for C57BL/6). After challenge, mice were observed daily to monitor their health and the appearance of any clinical signs. Disease severity was measured using the arbitrary scores described above. Each experiment was repeated 3 times. In all experiments, all animals were treated subcutaneously with Depo-Provera seven days before intravaginal inoculation and challenge, as described in the previous paragraph. At sacrifice, mice were anaesthetized intraperitoneally with 100 µl of isotonic solution containing 1 mg of Zoletil (Virbac, Milan, Italy) and 200 µg Rompun (Bayer, Milan, Italy), and blood, vaginal lavages and spleens were harvested. The two-tailed Mann-Whitney and Kaplan-Meier tests were used for the subsequent statistical analysis.

### Immunization with recombinant HSV1-LacZ with or without Tat protein and challenge with wild-type HSV1

The HIV-1 Tat protein (HTLV-IIIB isolate, BH10 clone), provided by Diatheva, was produced in *E. coli*, as previously described [70]. For *in vivo* experiments the Tat protein was diluted in saline buffer containing 1% sucrose and 1% human serum albumin as previously described [44]. Eight-week old C57BL/6 female mice were treated intravaginally with 10^3^ pfu/mouse of HSV1-LacZ, alone or in combination with 5 µg/100 µl of Tat protein, given intradermally. At day 28, mice (n = 5 per experimental group) were challenged intravaginally with a lethal dose (2×10^8^ pfu/mouse) of wild-type HSV1 (LV strain). After challenge, mice were observed daily to monitor their health and the appearance of any clinical signs. Disease severity was measured using the arbitrary scores described above. The Mann-Whitney test was used for statistical analysis of the results.

### Sample collection

Blood samples were incubated for 16 h at 4°C, centrifuged for 10 min 10,000 rpm (*9,877*×*g*) to obtain sera, and stored at −80°C until analysis. Samples of vaginal secretions were obtained by washing the vaginal cavity with 250 µL of PBS containing 5% FBS and a cocktail of protease inhibitors (Roche, Mannheim, Germany), and then incubated on ice for 1 h. Vaginal secretion samples were centrifuged at 10,000 rpm (*9,877*×*g*) in an Eppendorf microfuge for 10 min at 4°C. They were then treated with 5 µL of 1 M PMSF and 5 µL of 1% NaN_3_, and then stored at −20°C until analysis. Splenocytes were purified from spleens squeezed on filters (Cell Strainer, 70 µm, Nylon, Becton Dickinson, Milan, Italy). Following red blood cell (RBC) lysis with RBC lysing buffer (Sigma-Aldrich), cells were washed with RPMI 1640 (Invitrogen) containing 10% FBS (Hyclone, Invitrogen), spun for 10 min at 1,700 rpm (*557*×*g*) in a benchtop centrifuge, and cultured in complete RPMI 1640 medium.

### Vaginal lavage and titration

After immunization and challenge, infection was monitored at 1 and 3 days post-inoculation. Briefly, PBS (50 µl) was pipetted in and out of the vagina 10 times, diluted to 0.2 ml in completed medium, and titrated onto Vero cells in six-well plates, as described above. Three to four dilutions were prepared for each lavage. When infected cells (foci) were detected, the cells were fixed with 2 ml/well of 1% crystal violet solution, and the plaques were then counted as previously described [56]. The Kruskal-Wallis test was used for statistical analysis of the results.

### ELISpot assays

IFN-γ or IL-4 ELISpot assays were carried out using the murine kits provided by Becton Dickinson, according to the manufacturer’s instructions. Briefly, nitrocellulose plates were coated with 5 µg/ml of anti-IFN-γ or anti-IL-4 mAb for 16 h at 4°C. Plates were then washed with PBS and blocked for 2 h at 37°C with RPMI 1640 supplemented with 10% FBS. Total splenocytes freshly isolated from individual C57BL/6 mice (5×10^5^ cells/well) were plated into nitrocellulose-coated plates in the presence of HSV1-derived peptides (10^−6^ M) in complete RPMI 1640 medium for 24 h at 37°C. Total splenocytes from individual BALB/c mice (3×10^6^ cells) were re-stimulated *in vitro* with a pool of peptides (1 µg/ml each) and IL-2 (10 U/ml) for 5 days. After *ex vivo* restimulation, cells were washed with RPMI 1640 containing 10% FBS, and 5×10^5^ cells/well were plated into nitrocellulose-coated ELISpot plates, as described above. Cells incubated with 5 µg/ml concanavalin A (positive control) or with medium alone (negative control) were used as controls. Spots were quantified using an AELVIS 4-Plate ELISpot Reader (TEMA Ricerca, Bologna, Italy). The specific response was taken as the number of spots counted in the peptide-treated cultures minus the number of spots counted in the untreated cultures. Results are expressed as number of spot-forming units (SFU)/10^6^ cells. Values at least 2-fold higher than the mean number of spots in the control wells (untreated cells), i.e., ≥50 SFU/10^6^ cells, were considered positive. The two-tailed Mann-Whitney test was used for statistical analysis of the data.

### Analysis of antibody responses

Anti-HSV1 specific antibody titers (IgM, IgG, IgG_1_ and IgG_2a_) were measured by ELISA in serum samples collected from individual mice and plated in 96-well immunoplates (Nunc MaxiSorp, Milan Italy), previously coated with 100 ng/well of HSV1 purified viral lysate (MacIntyre Strain, Tebu-bio, Milan, Italy), and resuspended in PBS containing 0.05% NaN_3_, for 16 h at 4°C. Plates were washed five times with PBS containing 0.05% Tween 20 buffer (Sigma-Aldrich) using an automated washer (BioRad), and then blocked for 90 min at 37°C by the addition of 200 µl/well PBS containing 0.5% milk and 0.05% NaN_3_. After extensive washing, 100 µl/well of the appropriate dilutions of each serum were dispensed into duplicate wells and then incubated for 90 min at 37°C. Plates were washed again before the addition of 100 µl/well of HRP-conjugated goat anti-mouse IgG (Sigma-Aldrich), diluted 1∶1,000, or HRP-conjugated goat anti-mouse IgM (Sigma-Aldrich), diluted 1∶7,500, in PBS containing 0.05% Tween 20 and 1% BSA, and incubated at 37°C for 90 min. In each plate, two wells were incubated with PBS containing 0.5% milk, 0.05% NaN_3_ and the secondary antibodies (blank). The anti-HSV1 IgG isotype was detected using a goat anti-mouse antibody directed against IgG_1_ or IgG_2a_ (Sigma-Aldrich) diluted 1∶30,000 in PBS containing 0.05% Tween 20 and 1% BSA. After incubation, plates were washed five times and subsequently supplemented with a solution of 2,2′-azinobis [3-ethylbenzothiazoline-6-sulfonic acid]-diammonium salt (ABTS) substrate (Roche). The reaction was stopped after 50 min by adding 0.1 M citric acid. The absorbance values were measured at 405 nm using an automatic plate reader (Sunrise Tecan, Salzburg, Austria). The cut-off value was estimated as the mean optical density (OD) of 3 negative control sera plus 0.05. Each OD value was subtracted from the blank and cut-off values to obtain a net OD value. IgG titers were calculated using the Excel program intercept function. The Fisher exact test was used for statistical analysis of the results.

For IgA analysis, 96-well MaxiSorp plates were coated for 16 h at 4°C with 100 ng/well of HSV1 viral lysate to measure antigen-specific IgA, and with 0.1 ml of a goat anti-mouse IgA serum (0.2 mg/ml in PBS containing 0.05% NaN_3_; Sigma-Aldrich) to measure total IgA and to generate a standard curve. Plates were washed six times with washing buffer and incubated for 1 h at 37°C with PBS containing 1% BSA and 0.1% Tween 20. Plates were drained and samples tested (duplicate wells) starting from a 1∶20 dilution for total IgA, and 1∶8 dilution for antigen-specific IgA. To generate the standard curve, mouse IgA (Sigma-Aldrich) was tested in increments from 1.5 ng/ml to 100 ng/ml. After 1 h at 37°C, wells were washed six times, incubated with a goat biotin-conjugated anti-mouse IgA serum (Sigma-Aldrich) for 1 h at 37°C, then washed and incubated with HRP-conjugated streptavidin (Becton Dickinson) for an additional 30 min at 37°C. ABTS substrate was added, and colour development measured after 10 min incubation at 37°C, as described above. All plates included positive and negative control samples. The amount of antigen-specific IgA is expressed as a percentage (%) of specific IgA with respect to the total amount of IgA in the same sample.

### Evaluation of HSV1-Tat and HSV1-LacZ infection in CB1 mouse dendritic cells, and immunofluorescence

CB1 mouse dendritic cells (1×10^6^) were infected with HSV1-Tat or HSV1-LacZ at either 1 or 5 MOI. Infected cells were harvested 24 h post-infection and fixed with 4% paraformaldehyde for 20 min at room temperature. They were then rinsed twice in PBS, and incubated for 5 min in glycine 1%. Afterwards, cells were permeabilized for 5 min with 0.3% Triton 100X in PBS, rinsed twice with PBS, and blocked for 1 h with 10% BSA in PBS at room temperature. Cells were then incubated with a rabbit anti-herpes simplex polyclonal antibody (Accurate Chemical & Scientific Corporation, AXL237) diluted to 1∶1,000 in PBS (pH 7.2), rinsed three times with PBS, and incubated for 1 h at room temperature with a R-phycoerythrin-conjugated donkey anti-rabbit secondary antibody (Bethyl Laboratories, Inc.) diluted to 1∶100 in PBS. Cells were rinsed three times with PBS, and 4′,6-diamidino-2-phenylindole (DAPI) was added 5 min before the end of the procedure to stain the nuclei. Cells were examined under a Zeiss fluorescence microscope, pictures were taken using a digital camera, and images were processed using Adobe Photoshop.

### Proteasome purification and enzyme assay

CB1 mouse dendritic cells (2×10^6^) were infected with either HSV1-Tat or HSV1-LacZ at 5 MOI, and collected after 6, 12 or 24 h post-infection. Cells were washed with cold PBS and resuspended in 50 mM Tris-HCl (pH 7.5), 5 mM MgCl_2_, 1 mM dithiothreitol (Sigma-Aldrich), 2 mM ATP and 250 mM sucrose. Glass beads equivalent to the volume of the cell suspension were added, and the mixture was vortexed for 2 min at 4°C. Beads and cell debris were removed by centrifugation at 5,000 rpm (*2,000*×*g*) for 7 min, followed by centrifugation at 14,000 rpm (*13,500*×*g*) for 20 min. Lysates were cleared by 1 h of ultracentrifugation at 55,000 rpm (*100,000*×*g*), and supernatants were then ultracentrifuged at 55,000 rpm (*100,000*×*g*) for 5 h. Proteasome-containing pellets were re-suspended in 0.5 ml homogenization buffer [50 mM Tris-HCl (pH 7.5), 100 mM KCl, 15% glycerol]. Protein concentration was determined using the bicinchoninic acid protocol (Pierce, Rockford, IL). The chymotrypsin-like and trypsin-like activities of purified proteasomes from infected and uninfected cells were tested using the fluorogenic substrates Suc-LLVY-AMC and Boc-LRR-AMC, respectively, in the presence or absence of the proteasome inhibitor 25 µM carbobenzoxy-L-leucyl-L-leucyl-L-leucinal (MG-132, Sigma), as previously described [41]. Fluorescence was determined using a fluorimeter (Tecan), and proteasome activity was expressed as arbitrary fluorescence units (AFU). The two-tailed Wilcoxon signed-rank test was used for statistical analysis of the results.

## Results

### Construction and characterization of live attenuated HSV1 vectors and analysis of Tat expression

**Figure 1:**
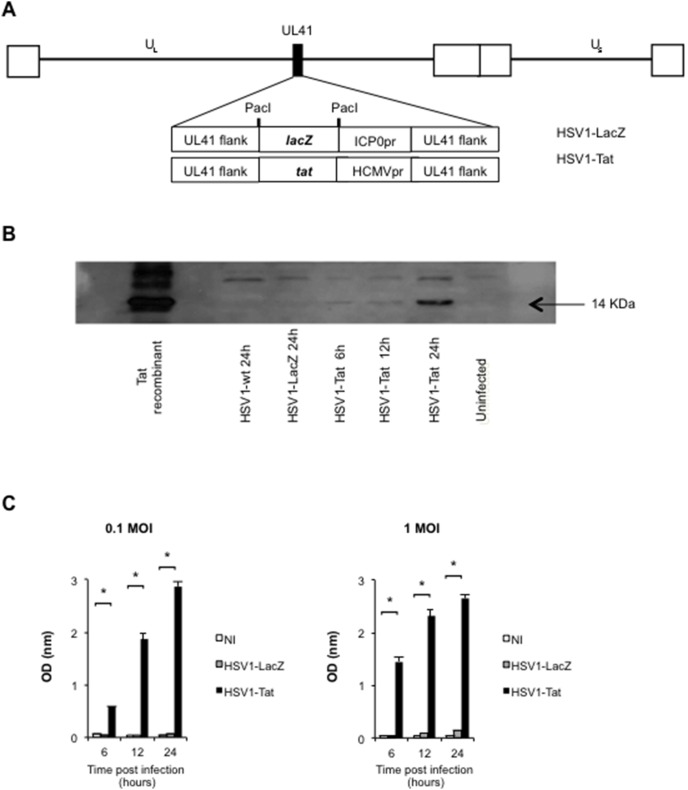
Vector representation and analysis of biological Tat expression. Schematic representation of the HSV1-LacZ/HSV1-Tat recombinant viruses (A). The black square in the HSV1 backbone indicates the UL41 locus (vhs gene), which has been deleted by insertion of the *lacZ-* or Tat-promoter cassette, respectively. The white squares represent the terminal and internal repeats of the HSV genome delimiting the unique regions (U_L_: unique long; U_S_: unique short). Analysis of Tat expression by HSV1-Tat recombinant vector (B). Western blot analysis of BALB/c cells infected with HSV1-Tat (1 MOI) after 6, 12 and 24 h post-infection. Negative controls are represented by uninfected cells and by cells infected with wild-type HSV1 or HSV1-LacZ. Recombinant Tat protein (Tat) was used as positive control. Analysis of biological Tat activity by CAT ELISA assay (C). CAT expression induced by functional Tat protein produced by the HSV1-Tat viral vector was measured 6, 12 and 24 h post-infection in HeLa3T1 cell extracts infected with HSV1-Tat (0.1 or 1 MOI). Negative controls were uninfected cells and cells infected with wild-type HSV1-LacZ. The means (± SD) of six independent experiments are shown.

Two live attenuated HSV1 vectors (Figure 1A), one containing the *lacZ* gene (HSV1-LacZ) and one the HIV-1 *tat* gene (HSV1-Tat) in the UL41 non-essential locus of the HSV1 genome strain LV [55], were constructed by means of a two-step method [54]. First, the HSV1-LacZ virus was generated by homologous recombination between wild-type HSV1 and the pB41-lacZ plasmid containing the *lacZ* marker gene, under the control of the ICP0 immediate early promoter of HSV inserted in the UL41 gene of HSV1 [53], [54]. Next, the HSV1-Tat recombinant virus was generated by homologous recombination between the pB41-HCMVtat plasmid and the viral HSV1-LacZ DNA, as detailed in the Methods section. The presence of *lacZ* and *tat* genes in the UL41 locus of both recombinant viruses was confirmed by Southern blot analysis (data not shown).

The expression of the Tat protein was assessed by Western blot analysis (Figure 1B). To this end, BALB/c fibroblast cell lines were infected with HSV1-Tat, and Tat expression was analysed at 6, 12 and 24 h post-infection. Uninfected cells and cells infected with wild-type HSV1 and HSV1-LacZ viruses were used as negative controls, and the recombinant Tat protein was used as positive control. As shown in Figure 1B, Tat expression was promptly detected at 12 h post-infection. Similar results were observed in Vero cells (data not shown).

Next, we determined whether the HSV1-Tat vector retained the biological activity of Tat. To this aim, HeLa3T1 cells [71], in which expression of the CAT reporter gene is driven by the HIV LTR and therefore only occurs in the presence of bioactive Tat protein, were infected with the live attenuated HSV1-Tat or HSV1-LacZ vectors at two different MOI (0.1 or 1). CAT expression was measured by ELISA 6, 12 and 24 h post-infection and, as shown in Figure 1C, it was readily detected by ELISA at 12 h after infection, even at the lowest MOI of 0.1, in cells infected with HSV1-Tat, but not in cells infected with HSV1-LacZ or in uninfected cells, demonstrating that the recombinant HSV1-Tat vector expresses a functionally active Tat protein generated by the recombinant HSV1-Tat.

The efficiency of HSV1-Tat and HSV1-LacZ replication was evaluated and compared in tissue culture. To this end, Vero cells were infected, either in monolayer or in suspension, with either 0.1 or 1 MOI of wild-type HSV1, HSV1-Tat or HSV1-LacZ, and the viral yield from each infection was determined after 18 h by the plaque assay titration method. As shown in Table 1, both viruses replicated with the same efficiency, as no impairment in viral infectivity or viral yield was observed between the two recombinant viruses.

**Table 1:** Evaluation of HSV1-Tat and HSV1-LacZ viral yields in vitro^a^.

Virus	Vero cells infected in suspension		Vero cells infected in monolayer	
	1 MOI	0.1 MOI	1 MOI	0.1 MOI
**HSV1-LacZ**	1.5×10^8^ pfu/ml	3.5×10^7^ pfu/ml	1×10^8^ pfu/ml	2.5×10^7^ pfu/ml
**HSV1-Tat**	1.2×10^8^ pfu/ml	3.7×10^7^ pfu/ml	8×10^7^ pfu/ml	3.2×10^7^ pfu/ml
**HSV1- wt**	1.9×10^8^ pfu/ml	1.8×10^8^ pfu/ml	2×10^8^ pfu/ml	1.2×10^8^ pfu/ml

a The replication of wild-type HSV1, HSV1-Tat and HSV1-LacZ viruses was evaluated *in vitro* using Vero cells (1×10^6^) infected, in monolayer or in suspension, with each virus at 0.1 or 1 MOI. Infected cells were harvested at 18 h post-infection, and viral production was assayed by means of the plaque assay method. Yields are expressed as the means of two independent experiments.

**Figure 2:**
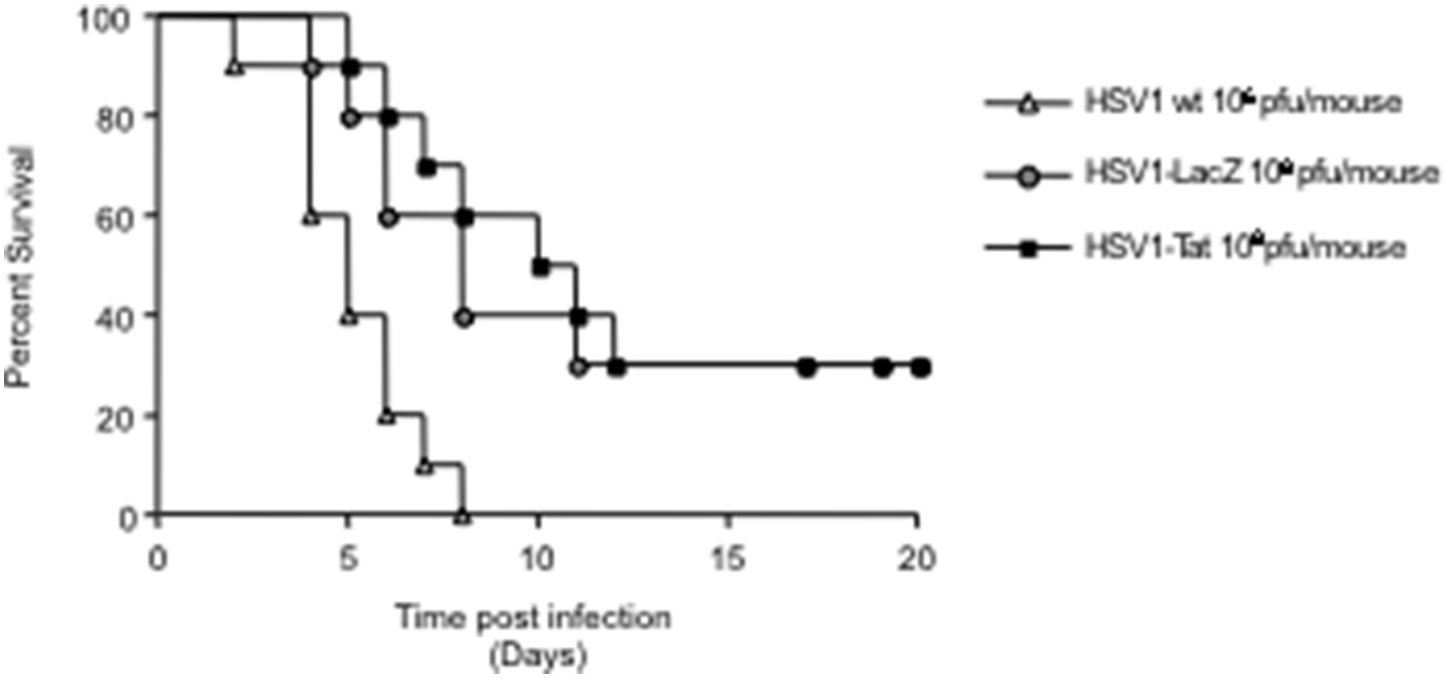
Determination of HSV1-Tat and HSV1-LacZ viral attenuation in Balb/c mice. Groups of mice (n = 10) were infected by the intravaginal route with a single dose (ranging from 10^4^ to 10^8^ pfu/mouse) of HSV1-Tat or HSV1-LacZ at a single time point. Mice were observed daily for survival up to 20 days post-infection, for survival. The results of the group inoculated with 10^8^ pfu/mouse are shown. The results of one representative experiment (out of three) are shown. The Kaplan-Meier test was used to estimate the probability of clinical manifestation.

Finally, to evaluate and compare the replication efficiency and attenuation of HSV1-Tat and HSV1-LacZ *in vivo*, both viruses were tested at different infectious doses. Groups of BALB/c mice were infected with a single intravaginal dose (ranging from 10^4^ up to 10^8^ pfu/mouse) of wild-type HSV1, HSV1-Tat or HSV1-LacZ, and each group was monitored for survival and the appearance of any signs of disease for up to 20 days post-infection. Both vectors behaved similarly and showed the same degree of attenuation at each infectious dose (data not shown). Remarkably, as shown in Figure 2, at 20 days after infection 20%–30% of mice infected with HSV1-Tat and HSV1-LacZ at the dose of 10^8^ pfu/mouse were still alive, whereas 100% of mice succumbed to infection with wild-type HSV1 at 10^6^ pfu/mouse. Altogether these results demonstrate that HSV1-Tat and HSV1-LacZ vectors infect and replicate in a similar fashion, both *in vitro* and *in vivo,* with comparable attenuation.

### Analysis of protection against lethal infection with wild-type HSV1 after immunization of mice with live attenuated recombinant HSV1 vectors

**Figure 3:**
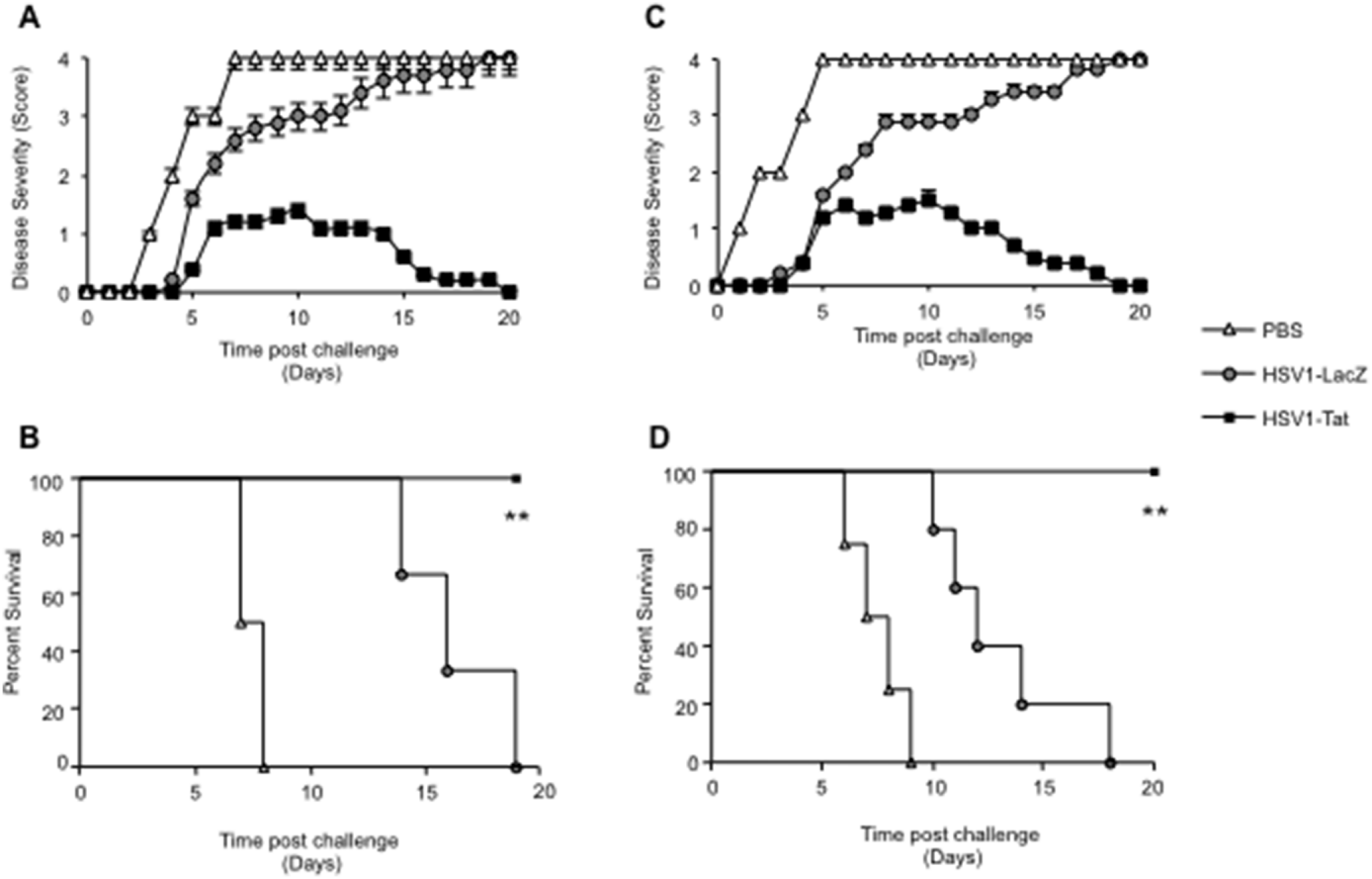
Disease severity and survival of BALB/c (A and B) or C57BL/6 (C and D) mice inoculated intravaginally with HSV1-LacZ or HSV1-Tat after challenge with a lethal dose of wild-type HSV1. Ten mice/group were inoculated intravaginally with 10^3^ pfu/mouse of HSV1-LacZ, HSV1-Tat or control buffer, and, 28 days later, challenged intravaginally with a lethal dose of wild-type HSV1 (2×10^6^ pfu/mouse for BALB/c mice and 2×10^8^ pfu/mouse for C57BL/6 mice). Mice were observed daily for appearance of signs of HSV1 disease and death. Disease severity scores (A for BALB/c and C for C57BL/6) and survival (B for BALB/c and D for C57BL/6) were assessed. Mean disease scores (± SD) for each group is shown. The results of one representative experiment (out of three) are shown. Data were analysed statistically using the two-tailed Mann Whitney test (panels A and C) and the Kaplan-Meier test. **P<0.01 (panels B and D).

To investigate the potential use of the two attenuated HSV1 vectors as anti-HSV vaccines, groups of female BALB/c and C57BL/6 mice were given a single intravaginal immunization of either HSV1-Tat or HSV1-LacZ at 10^3^ pfu/mouse. Control mice received PBS alone. At day 28, all mice were challenged intravaginally with a lethal dose of wild-type HSV1, and monitored for signs of disease and survival. As shown in Figure 3, all control C57BL/6 and BALB/c mice had developed severe disease (score 3) 4–5 days after challenge, and had died by day 5 (C57BL/6) or 7 (BALB/c) without showing any sign of recovery. In sharp contrast, all C57BL/6 and BALB/c mice treated intravaginally with HSV1-LacZ had a disease score of <2 at day 5 after challenge, and at day 7 all of them were still alive, indicating that immunization with HSV1-LacZ provided some degree of protection against lethal infection. As all C57BL/6 and BALB/c mice immunized with HSV1-LacZ went on to develop severe HSV1 disease and died (100% mortality by day 18 post-infection), it was apparent that immunization with HSV1-LacZ had conferred only partial protection against challenge. More encouragingly, all mice immunized with HSV1-Tat presented only mild, transient signs of disease, and 100% of them survived the viral challenge, apparently making a complete recovery (Figure 3).

**Figure 4:**
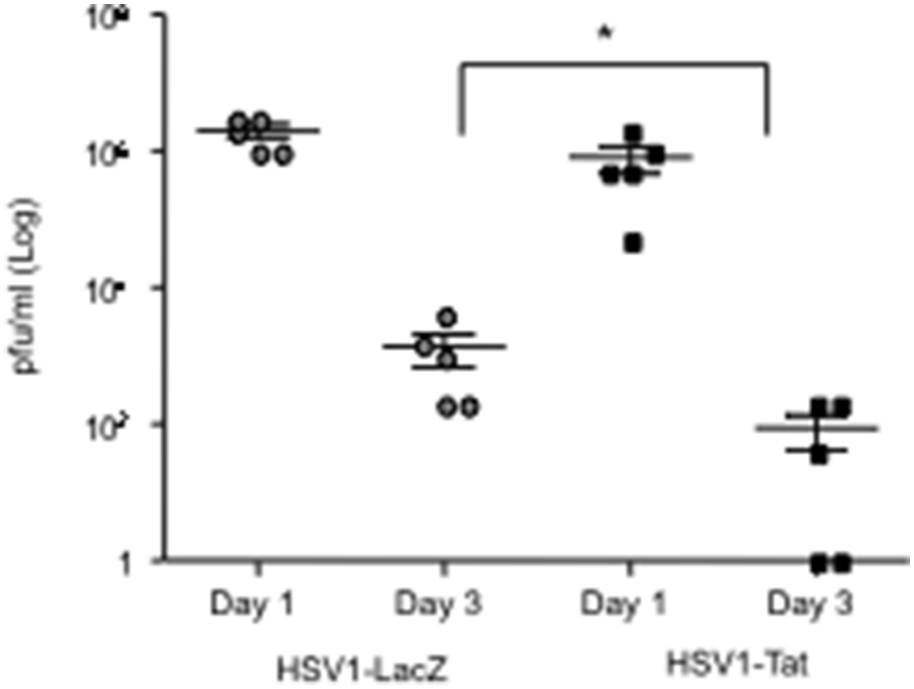
Vaginal lavages after lethal challenge with HSV1. C57BL/6 mice (n = 5) were immunized with 10^3^ pfu/mouse of HSV1-LacZ or HSV1-Tat and, 28 days later, challenged intravaginally with a lethal dose of wild-type HSV1 (2×10^8^ pfu/mouse). Vaginal lavages were collected at days 1 and 3 post-challenge, and HSV1 replication was monitored by virus titration onto Vero cells using the plaque-assay method. The Kruskal-Wallis test was used for statistical analysis, *p<0.07.

In line with these results, at day 3 after challenge, vaginal lavages of C57BL/6 mice immunized with HSV1-Tat showed lower viral titers than those observed in mice immunized with HSV1-LacZ (Figure 4), suggesting that immunization with HSV1-Tat contained viral replication in the vaginal lumen. However, it remains to be established whether HSV1-specific immune responses elicited by the presence of Tat within the vector could control viral replication in other organs including CNS.

### Analysis of protection against a lethal challenge with wild-type HSV1 in mice immunized with live attenuated recombinant HSV1-LacZ vector and Tat protein

**Figure 5:**
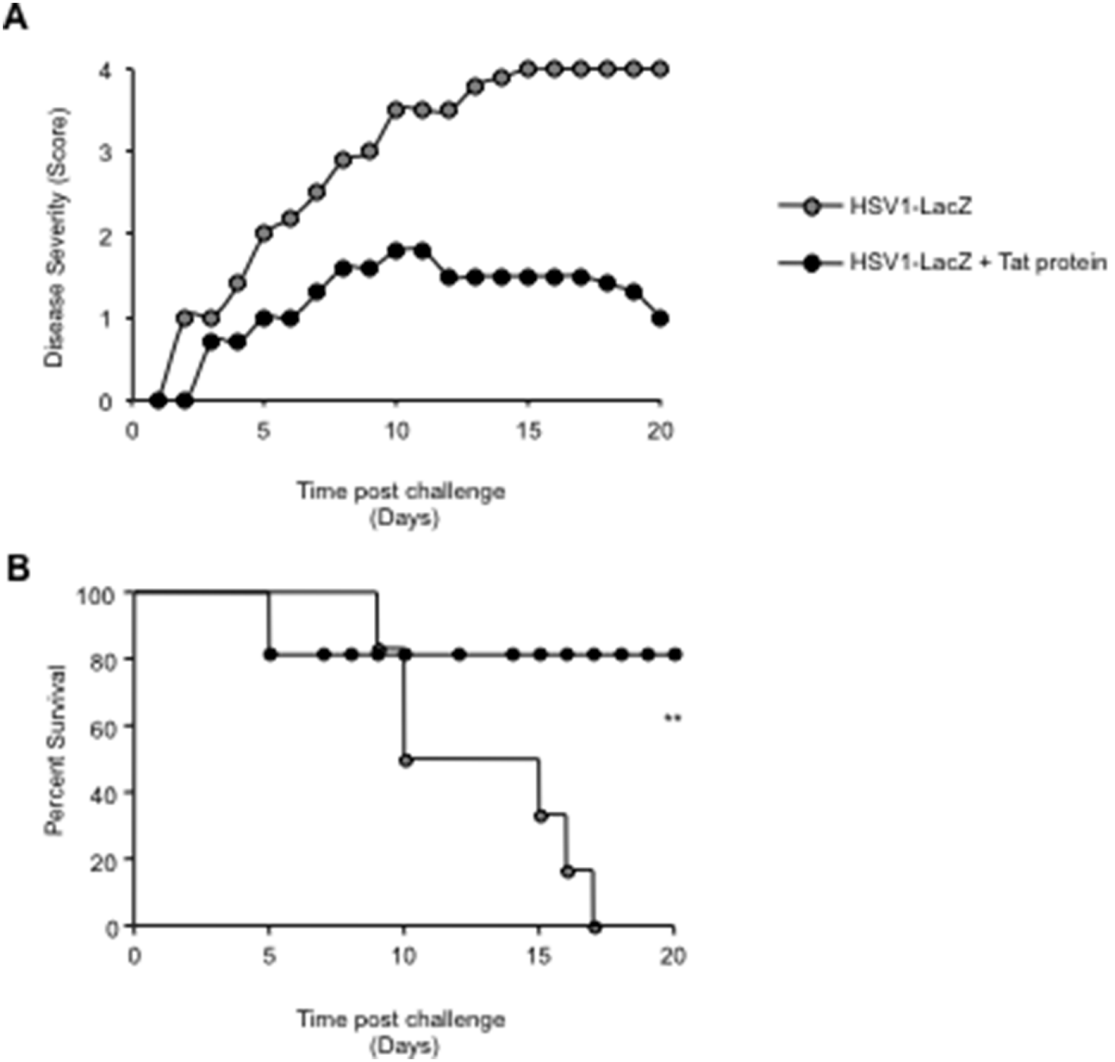
Analysis of the role of Tat protein on disease severity and survival of C57BL/6 mice. Mice (n = 5) were inoculated intravaginally with 10^3^ pfu/mouse of HSV1-LacZ and treated with (black dots) or without (grey dots) 5 µg/100 µl/mouse of Tat protein, administered intradermally at the same time. Control mice were inoculated with HSV1-LacZ alone. After 28 days mice were challenged intravaginally with a lethal dose of wild-type HSV1 and checked daily for the appearance of signs of disease (A) and survival (B). The two-tailed Mann-Whitney test was used for statistical analysis. **p<0.007.

To confirm the protective role of Tat, C57BL/6 mice were immunized intravaginally with 10^3^ pfu/mouse of the HSV1-LacZ vector, with or without 5 µg of biologically active Tat protein, given intradermally. At day 28, all mice were challenged intravaginally with the lethal dose of wild-type HSV1 and monitored for survival and the appearance of signs of the disease. As shown in Figure 5, all animals treated with the HSV1-LacZ virus and the Tat protein displayed significantly higher protection and survival than the group immunized with HSV1-LacZ alone, confirming the previous results indicating that the 100% protection observed in mice immunized with HSV1-Tat was due to the expression of the Tat protein.

### Analysis of HSV1-specific T-cell responses in mice infected with recombinant HSV1 vectors

To determine the effect of Tat expression on the induction of HSV1-specific T-cell mediated responses, female C57BL/6 and BALB/c mice were infected intravaginally with 10^3^ pfu/mouse of the attenuated replication-competent recombinant HSV1-LacZ or HSV1-Tat viruses. After 7 days, the presence of HSV1-specific T-cell responses in C57BL/6 mice was evaluated by IFN-γ and IL-4 ELISpot assays on fresh splenocytes. T-cell responses were evaluated using three K^b^-restricted CTL peptide epitopes, including the immunodominant SSIEFARL (SSI) and ITAYGLVL (ITA) epitopes, respectively derived from HSV1 glycoprotein B [57], [58] and glycoprotein K [59], [60], and the subdominant QTFDFGRL (QTF) epitope derived from ribonucleotide reductase 1 [61]. Similarly, T-cell responses in BALB/c mice were evaluated using two peptides, including the peptide SLKMADPNRFRGKDLP (SLK), which contains both the H^b^-restricted CD4 and K^d^-restricted CD8 immunodominant epitopes derived from glycoprotein D [64]–[66], and the CTL subdominant epitope DYATLGVGV (DYA), derived from ICP27 [62], [63].

**Figure 6:**
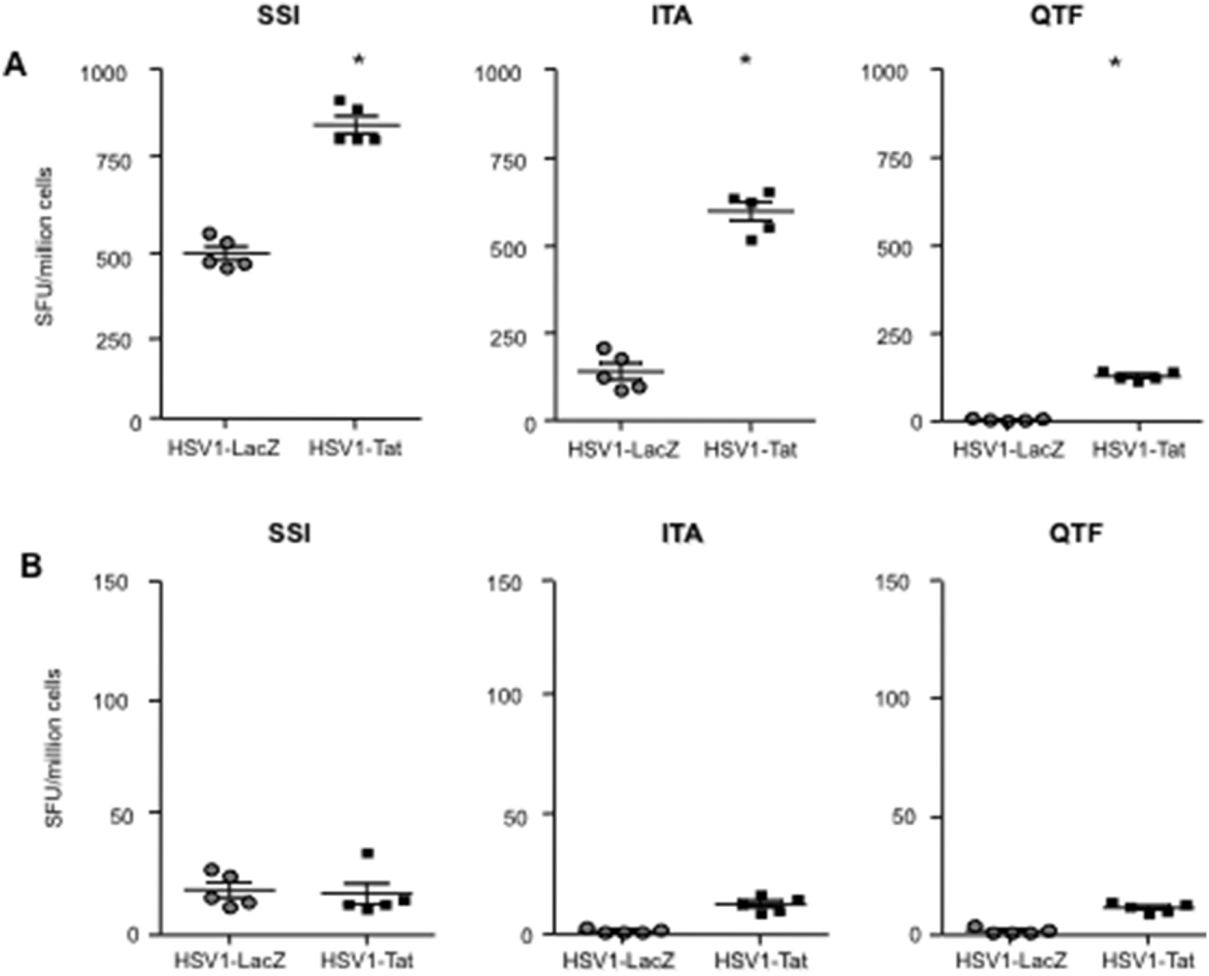
Analysis of HSV1-specific T-cell responses in individual C57BL/6 mice. Mice were inoculated intravaginally with 10^3^ pfu/mouse of live attenuated HSV1-Tat or HSV1-LacZ. Seven days after infection, splenocytes were isolated from 5 mice/group and tested by ELISpot assay for IFN-γ (A) or IL-4 (B) cytokine production upon stimulation with SSI, ITA and QTF HSV-derived peptides. Results are expressed as number of spot-forming units (SFU)/million cells per mouse. Values at least 2-fold higher than the mean number of spots in the control wells (untreated cells), i.e., ≥50 SFU/million cells, were considered positive. The results of one representative experiment (out of three) are shown. The two-tailed Mann-Whitney test was used for statistical analysis, *p<0.05.

As shown in Figure 6A, splenocytes from C57BL/6 mice infected with HSV1-LacZ released IFN-γ in response to the immunodominant SSI and ITA CTL epitopes, but not to the subdominant QTF epitope. In contrast, mice infected with HSV1-Tat responded to all three epitopes, including the subdominant QTF epitope, and, remarkably, responses to the immunodominant SSI and ITA peptides were significantly higher than those seen in control mice (Figure 6A). Th2-type responses, as measured by IL-4 release, were absent in both groups of mice (Figure 6B). These results demonstrate that the presence of Tat in an attenuated HSV1 vector increases CTL responses directed to HSV1, and promotes the induction of CTL responses against a subdominant epitope that are absent in C57BL/6 mice immunized with the HSV1-LacZ vector.

**Figure 7:**
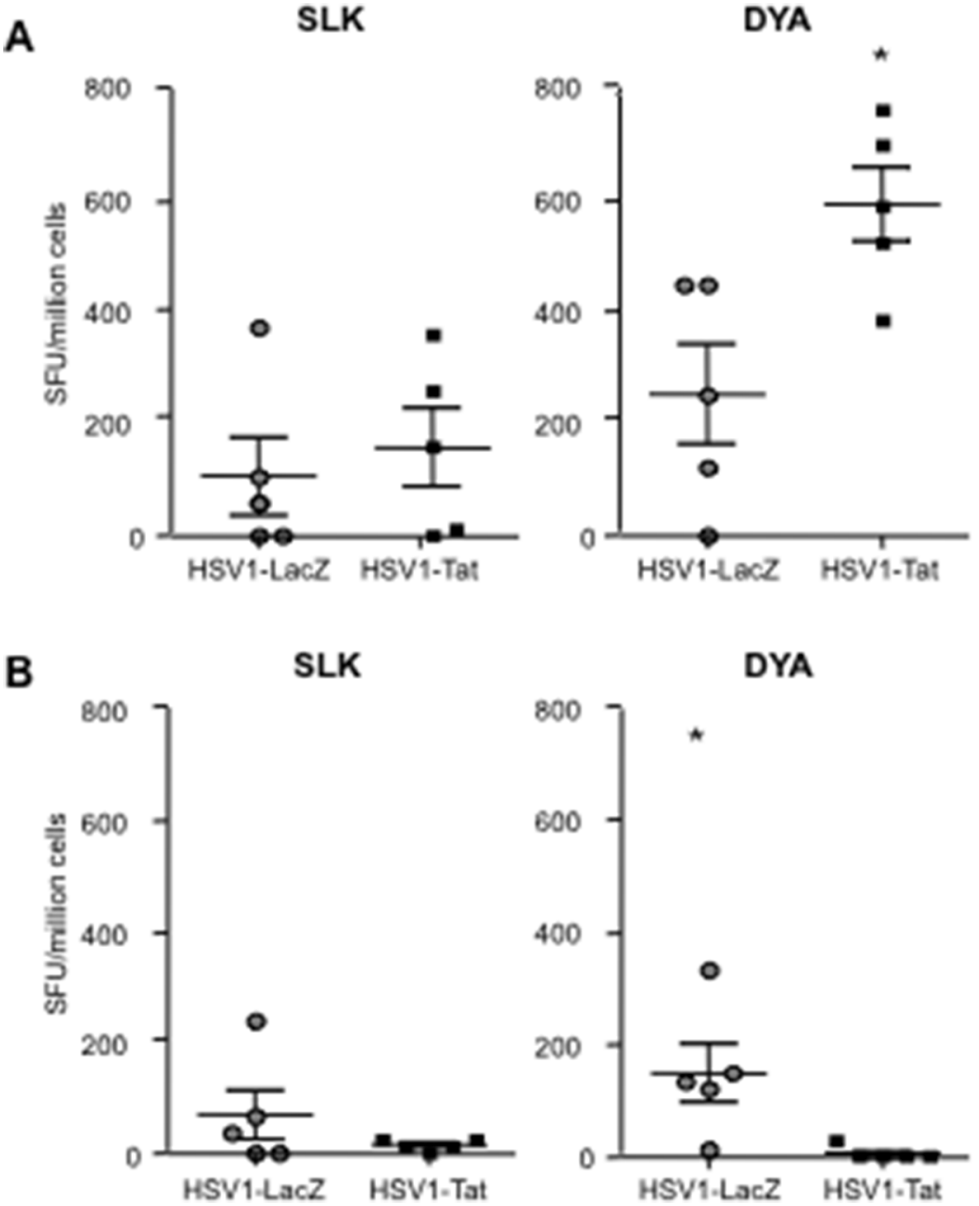
Analysis of HSV1-specific T-cell responses in individual BALB/c mice. Mice were inoculated intravaginally with 10^3^ pfu/mouse of live-attenuated HSV1-Tat or HSV1-LacZ. Seven days after infection splenocytes were isolated from 5 mice/group, stimulated for 5 days with the SLK and DYA HSV-derived peptides, and then assessed by ELISpot for IFN-γ (A) or IL-4 (B) cytokine production. Results are expressed as number of spot-forming units (SFU)/million cells per mouse. Values at least 2-fold higher than the mean number of spots in the control wells (untreated cells) and ≥50 SFU/million cells were considered positive. The results of one representative experiment (out of three) are shown. The two-tailed Mann-Whitney test was used for statistical analysis. *P<0.05.

Since fresh splenocytes from BALB/c mice responded poorly to HSV1 peptide epitopes (data not shown), they were re-stimulated *in vitro* for 5 days with the peptides and then assayed by IFN-γ and IL-4 ELISpot. As shown in Figure 7, in response to SLK [64]–[66] and DYA [62], [63] peptides, splenocytes from mice infected with HSV1-LacZ released IFN-γ (Figure 7A) and IL-4 (Figure 7B), while mice infected with HSV1-Tat released IFN-γ but not IL-4. Interestingly, the IFN-γ response to the subdominant DYA peptide was significantly higher in mice immunized with HSV1-Tat (Figure 7A). Together these results demonstrate that the intravaginal immunization of two different strains of mice with a recombinant HSV1 vector expressing Tat induced stronger HSV1-specific CTL responses, especially against subdominant epitopes, than immunization with HSV1-LacZ. However, it should be noted that in the case of BALB/c mice, the *in vitro* restimulation *per se* promoted preferential expansion of T cells responding to the subdominant DYA peptide, as compared to the dominant SLK epitope. Nevertheless, the expansion of DYA-specific T cells was much more pronounced in mice receiving HSV1-Tat than in mice infected with HSV1-LacZ, highlighting the ability of Tat to modulate the T cell repertoire (Figure 7A).

### Analysis of HSV1-specific humoral response in mice immunized with recombinant HSV1 vectors

**Figure 8:**
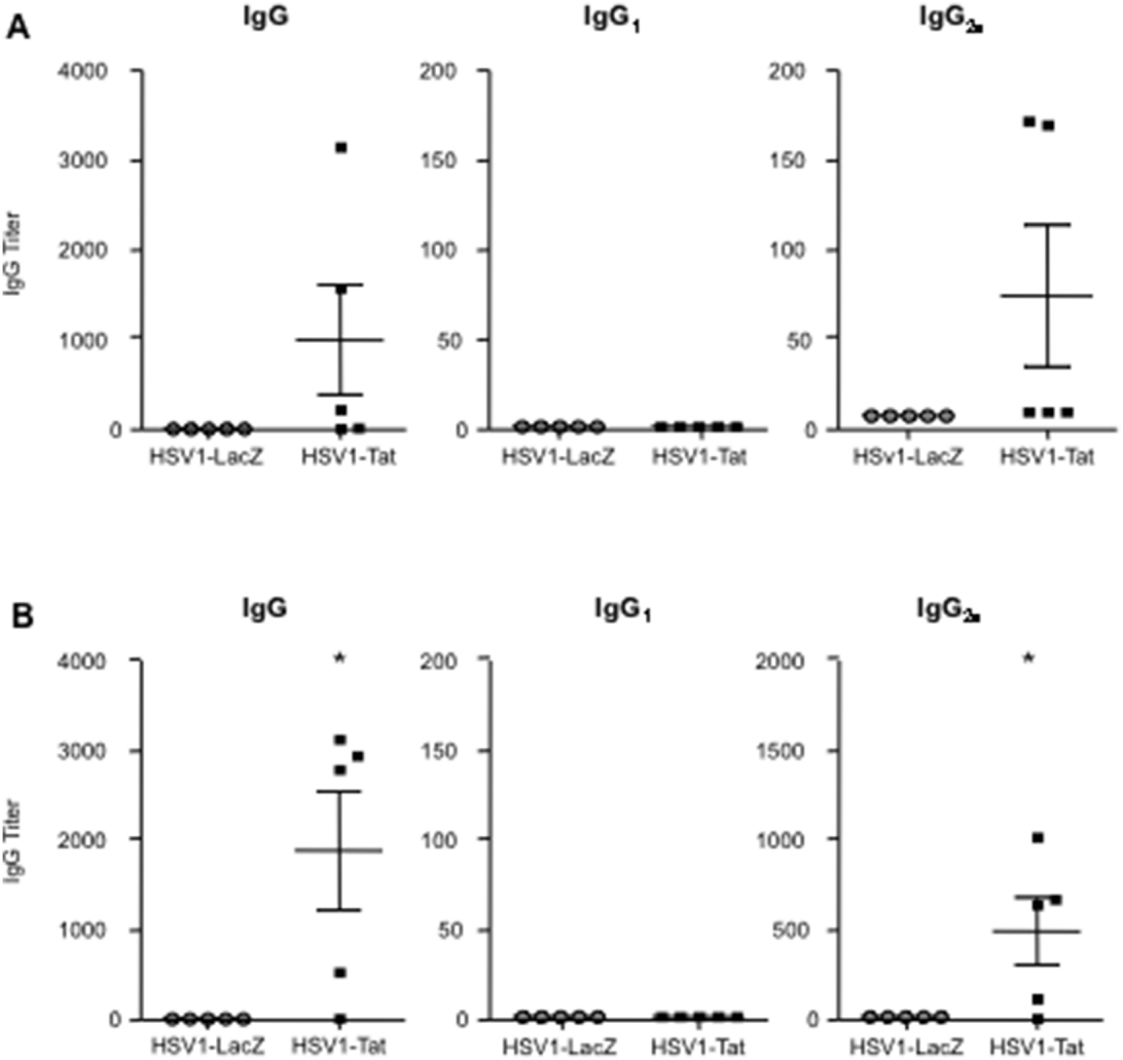
Evaluation of anti-HSV1 specific antibody titers (IgG, IgG_1_, and IgG_2a_) in sera from C57BL/6 (A) or BALB/c (B) mice treated by the intravaginal route with 10^3^ pfu/mouse of HSV1-Tat or HSV1-LacZ. Twenty days after infection, sera from 5 mice/group were collected by eye-bleeding, and anti-HSV1 antibody responses were measured by ELISA. The results of one representative experiment (out of three) are shown. The Fisher exact test was used for statistical analysis. *P<0.05.

We then evaluated the capacity of recombinant HSV1 vectors to induce antibodies against HSV1. To this end, sera and vaginal washes from C57BL/6 and BALB/c mice were collected at days 20 (Figure 8) and 27 (Figure S1) after intravaginal immunization with 10^3^ pfu/mouse of HSV1-Tat or HSV1-LacZ, and assessed by ELISA for the presence of anti-HSV1 IgM, IgG, and IgA. Surprisingly, as shown in Figures 8 and S1, irrespective of the mouse strain, intravaginal immunization with HSV1-Tat induced the generation of HSV1-specific serum IgG in the majority of the mice, whereas such responses were not detected in mice immunized with HSV1-LacZ. The IgG isotypes were further analysed, revealing the presence of IgG_2a_, but not IgG_1_, antibodies, indicating a Th1-type immune response (Figures 8 and S1). In this experimental setting, we did not detect the presence of either IgA or IgM in the sera, nor IgG or IgA in vaginal washes of mice immunized with the two HSV1-derived vectors (data not shown). These results demonstrate that intravaginal immunization of two different strains of mice with a recombinant HSV1 vector expressing Tat can promote the induction of HSV1-specific antibody responses (IgG_2a_ isotype), while immunization with a recombinant HSV1-LacZ vector cannot.

### Effect of recombinant HSV1 vectors on proteasome activities

**Figure 9:**
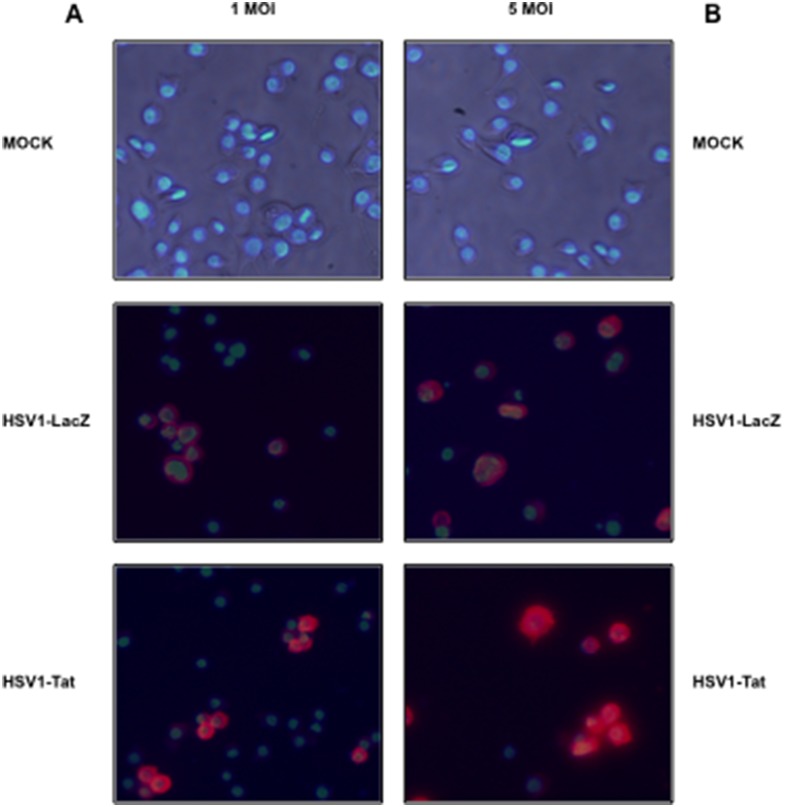
Immunofluorescence micrographs at 24-infection of CB1 cells mock-infected (A and B, top panels) or infected with 1 (A) or 5 (B) MOI of HSV1-LacZ (middle panels) or of HSV1-Tat (bottom panels). Reactivity with anti-HSV1 antibodies is shown in red whereas DAPI-labelled nuclei are shown in blue in all panels.

As the vast majority of CTL epitopes are generated by proteasomes, the effects of HSV1-Tat and HSV1-LacZ recombinant vectors on the activity of the proteasome were evaluated in mouse CB1 dendritic-like cells, to explore a possible mechanism underlying the increased and broadened immunogenicity of HSV1-Tat. The first step was to ascertain the ability of HSV1-Tat and HSV1-LacZ to efficiently infect CB1 cells, which were therefore incubated with either 1 or 5 MOI of HSV1-Tat or HSV1-LacZ vectors, and analysed by immunofluorescence microscopy using an anti-HSV polyclonal antibody. As shown in Figure 9, both HSV1-Tat and HSV1-LacZ infected cells to a similar extent.

**Figure 10:**
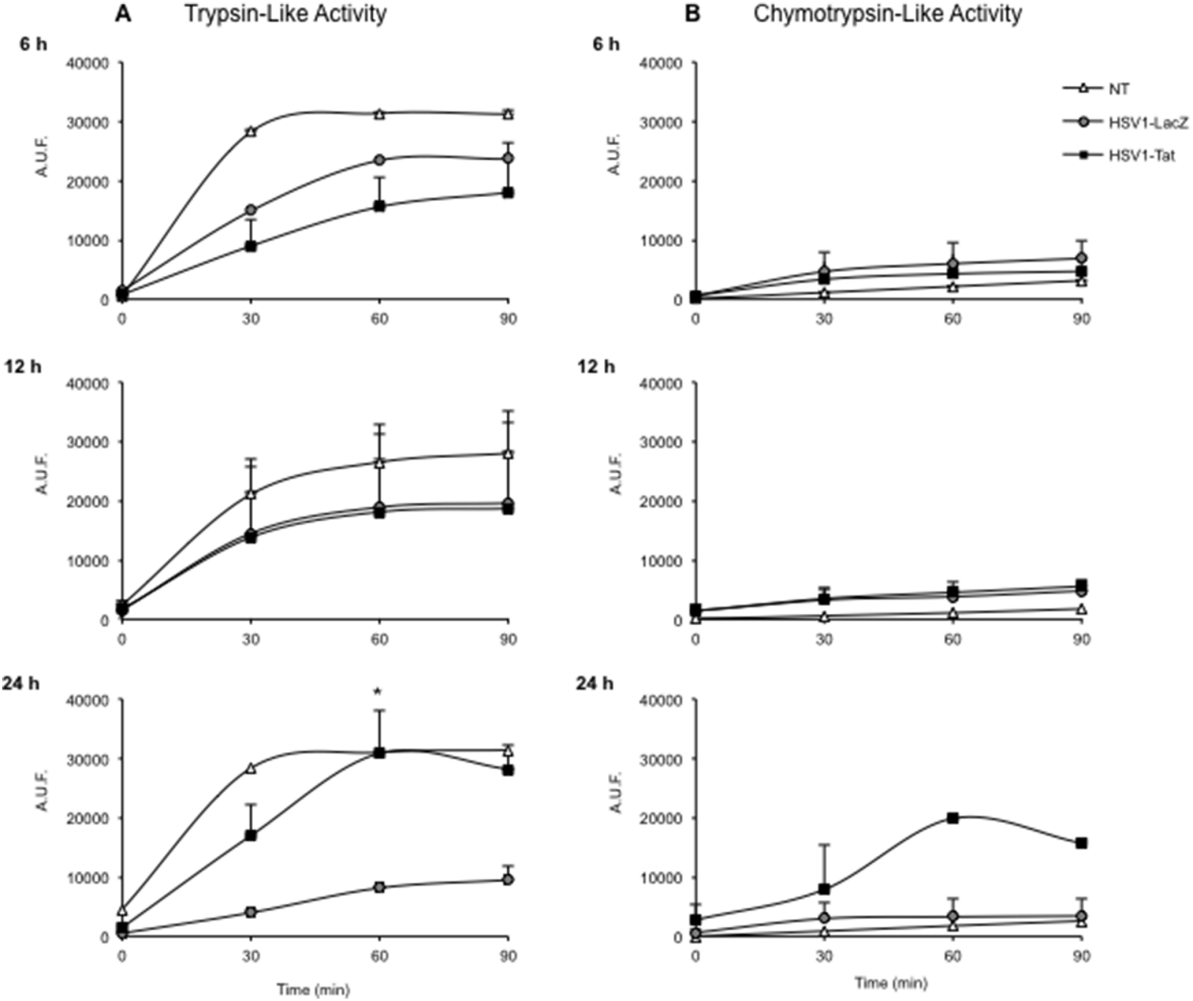
Proteolytic activity of proteasomes isolated from CB1 cells, uninfected or infected with 5 MOI of HSV1-Tat or HSV1-LacZ. Trypsin-like (A) and chymotrypsin-like (B) proteasome activities measured at 6, 12, and 24 h after infection are shown. Proteasome activity is expressed as arbitrary fluorescence units (AFU). The means (± SD) of three independent experiments are shown. The two-tailed Wilcoxon signed-rank test was used for statistical analysis, *p<0.05.

Next, equal amounts of proteasomes isolated from mouse CB1 dendritic-like cells, uninfected or infected with 5 MOI of HSV1-Tat or HSV1-LacZ vectors were analysed for trypsin-like and chymotrypsin-like activities at 6, 12, and 24 h after infection in the presence or absence of the proteasome inhibitor MG132. This revealed that, at 6 and 12 h post-infection, both vectors reduced the trypsin-like activity, as compared to uninfected cells (Figure 10A), whereas the chymotrypsin-like activity was comparable to that of untreated cells (Figure 10B). However, at 24 h post-infection, proteasomes purified from cells infected with HSV1-Tat had significantly higher trypsin-like (Figure 10A) and chymotrypsin-like (Figure 10B) activities than proteasomes purified from cells infected with HSV1-LacZ. All proteasomal acitivities were completely blocked by the presence of MG132 (data not shown). It therefore appears that the expression of Tat within a viral vector increases the proteolytic activities of the proteasome, in line with previous data obtained with the Tat protein alone [41], [72]. In this regard, the increased activity observed at 24 h post-infection (but not at earlier time points) is conceivably due to the time required for Tat to be expressed by the vector and exert its effects.

## Discussion

This study demonstrates that Tat co-expressed with HSV1 antigens by an attenuated replication-competent HSV1 vector impacts the course of HSV1 infection in mice by conferring full protection from symptoms arising from challenge with a lethal dose of wild-type virus in 100% of mice immunized with such a vector. The presence of Tat within the vector favored the induction of stronger and broader HSV1-specific humoral and cellular Th1-type immune responses, which may be responsible for the observed protection. In contrast, treatment with the HSV1-LacZ vector elicited weaker, narrower immune responses, and only delayed the appearance of disease signs, failing to protect mice from severe disease and death, which occurred in 100% of treated animals.

Notably, irrespective of the mouse model used, the presence of Tat within the HSV1 vector promoted the onset of cellular responses directed against subdominant HSV1-derived CTL epitopes that are absent (as in the case of the response against the QTF epitope in C57BL/6 mice) in mice immunized with HSV1-LacZ. This is in line with previous *in vivo* findings showing that Tat induces epitope-specific T-cell responses directed against subdominant and cryptic epitopes of heterologous antigens that are not detected in mice vaccinated in the absence of Tat [40]. As the previous finding were from preclinical studies aimed at developing a subunit vaccine against HIV based on the co-administration of Tat protein with the HIV-1 Gag or Env proteins, this is first time the immunomodulatory effect of Tat has been demonstrated using a viral vector expressing the *tat* gene.

Our results also show that the presence of Tat within the HSV1 vector increased the proteolytic activities of the proteasome (Figure 10), in all likelihood contributing to better generation and presentation of CTL epitopes and thus to the induction of broader, effective HSV-specific CTL responses. This is also in line with our previous observations demonstrating that the Tat protein alone affects the subunit composition and activity of immunoproteasomes, resulting in a better presentation of subdominant and cryptic CTL epitopes of heterologous antigens [41], [72].

It is now well recognized that CD4^+^ and CD8^+^ T-cell responses from asymptomatic HSV patients are different from those in symptomatic patients [24]–[26], [73], [74], indicating that they have an important role in containing HSV replication. This may explain the failure of vaccine trials conducted so far, and provides a rationale for the development of vaccines that promote T-cell responses against these different sets of asymptomatic epitopes. Furthermore, the increase in HSV1-specific T-cell responses induced by the HSV1-Tat vector may reflect Tat’s ability to modulate T-cell functionality. This is supported by several *in vitro* and *in vivo* studies demonstrating that the biologically active clade-B Tat protein modulates the various signals orchestrating the first phases of the immune response through enhancement of the co-stimulation provided by IL-2 [75], [76], CD40 [37], [38], CD28 [76] and other pro-inflammatory cytokines [77]–[81]. Moreover, Tat is known to target immature DCs, inducing their maturation towards a Th1-polarizing phenotype through the transcriptional activation of TNF-alpha gene expression, leading to more efficient presentation of both allogeneic and heterologous antigens [37], [38]. It is therefore plausible that both Tat-mediated modulation of antigen presentation and increased expansion of epitope-specific CTLs could contribute to the enhancement of T-cell responses directed against HSV.

In addition, Tat has been shown to up-regulate the expression of the transcription factor T-bet [82], which is crucial for HSV control [83], [84], and this may enable Tat to promote Th1 responses and class-switch recombination to the IgG_2a_ isotype in B cells [85], [86]. Consistent with these observations, our data clearly demonstrate that Tat induces enhancement of IFN-γ release – while dampening Th-2 responses against HSV antigens (Figure 6, 7) – and promotes the production of IgG_2a_ associated with Th1-type responses (Figure 8). This is particularly striking in BALB/c mice, a strain known to be prone to Th-2 responses [87].

Unexpectedly, our results also demonstrate, at least in this experimental framework, that immunization with the HSV1-LacZ vector does not induce the production of anti-HSV1 antibodies, whereas immunization with HSV1-Tat does induce HSV1-specific antibodies in some mice (Figure 8). As mentioned above, IgG responses elicited in mice vaccinated with HSV1-Tat were dominated by the IgG_2a_ subclass, and IgG_1_ is completely absent. In our opinion, this production of anti-HSV1 antibodies may depend on the general enhancement of IFN-γ release induced by Tat, although in different experimental settings it has been shown that Tat, due to its inherent adjuvanticity, promotes humoral responses against itself, as well as against unrelated antigens [42], [88].

Immunological data presented in this study show that HSV1-Tat is more immunogenic than HSV1-LacZ. Notably, it resulted in 100% protection from death after challenge with a lethal dose of wild-type HSV1 (Figure 3). This contrasts with HSV1-LacZ, which merely delayed death. Indeed, mice immunized with HSV1-Tat showed only mild signs of disease in the early stage, but these soon disappeared, highlighting the protective immunity elicited by this vector. Although we did not investigate the involvement of innate immunity, which is also known to play a role in the control of HSV infection [89], [90] and is, to some extent, modulated by Tat [91], [92], the data suggest that the observed protection is due to the capacity of the HSV1 vector expressing Tat to broaden and increase systemic HSV1-specific cellular responses. In addition, the immunization strategy described here also elicited local HSV1-specific T cells in the genital tract (data not shown). Though further studies are needed to quantify these cells and to define their phenotype, it is quite well established that local immunity [73], [93], [94] and broad T-cell responses [30], [94]–[97] are efficient in controlling HSV.

The role of humoral responses in the control of HSV infection is controversial. Indeed, the presence of natural or vaccine-induced HSV-specific antibodies has shown no appreciable effect on infection and reactivation [94], [97]–[101] in humans. Although other groups have demonstrated that the presence of HSV antibodies correlates with protection in animal model [98]. However, passive transfer of antibodies failed to protect the animals, suggesting that other immune responses, likely induced together with the antibody response, were actually responsible for the observed protection [98]. Also in our experimental conditions antibodies do not appear to be essential for protection. In fact, anti-HSV1 antibodies were not detected in the vaginal lavages of either HSV1-LacZ or HSV1-Tat immunized mice, and only few mice treated with HSV1-Tat presented serum anti-HSV1 IgG responses. This suggests that anti-HSV antibodies may be dispensable to protect against lethal challenge, as opposed to the broadened and increased cellular immune responses that were detected in all mice vaccinated with HSV1-Tat. This is in line with several evidences suggesting that CD8^+^ T cell responses are important for controlling HSV replication, and may play a role in halting viral reactivation from latency [20], [101]–[105].

It would therefore be useful to conduct future studies designed to further elucidate the role of Tat in modulating the response against HSV1 and, more importantly, to determine whether and to what extent immunization with HSV1-Tat reduces and controls the spread of the virus from the epithelium to the innervating neurons. If this is found to be the case, the establishment of latent infection within the dorsal root ganglia would be hampered, and the appearance and/or frequency of recurrent infections reduced.

In conclusion, the results of this study indicate that the immunomodulatory properties of Tat may be particularly relevant to vaccination strategies against intracellular pathogens, as already shown in studies aimed at developing a protective immunity against *Leishmania major* [45]. In particular, we suggest that Tat may be a suitable candidate to further investigate in the search for a new generation of HSV1-derived vectors with the capacity to induce broad HSV1-specific immune responses, and thereby control HSV1 infections.

## Supporting Information

Figure S1 (TIFF)
